# Bone health in cerebral palsy and introduction of a novel therapy

**DOI:** 10.1590/S1679-45082015AO3321

**Published:** 2015

**Authors:** Morton Aaron Scheinberg, Ricardo Prado Golmia, Adriana Maluf Elias Sallum, Maria Guadalupe Barbosa Pippa, Aline Pinheiros dos Santos Cortada, Telma Gomes da Silva

**Affiliations:** 1 Hospital Israelita Albert Einstein, São Paulo, SP, Brazil.; 2 Associação de Assistência à Criança Deficiente, São Paulo, SP, Brazil;; Lar Escola São Francisco, São Paulo, SP, Brazil.; 3 Associação de Assistência à Criança Deficiente, São Paulo, SP, Brazil.

**Keywords:** Cerebral palsy/drug therapy, Osteoporosis/drug therapy, Antibodies, monoclonal/therapeutic use, Child

## Abstract

**Objective:**

To assess the bone health status of children with cerebral palsy and the therapeutic effect of denosumab in a subgroup of children with cerebral palsy and decreased bone mass.

**Methods:**

Children with cerebral palsy were evaluated according to their motor disability score (classification system gross motor functions III to V), bone density and bone turnover markers. Dual X-ray energy absorption was used to measure the lumbar spine, and total body, except the head. Thereafter a group of children with cerebral palsy and osteoporosis was treated with denosumab, a fully human monoclonal antibody. Bone turnover markers were measured before and three months after treatment.

**Results:**

Reduction in bone mineral density was observed, particularly in children with greater impairment evaluated by the motor score. Decreased bone turnover markers were found in a selected group of children three months after exposure to denosumab.

**Conclusion:**

Bone loss was present in children with significant impairment of motor function, as well as decreased serum levels of bone resorption markers with new forms.

## INTRODUCTION

Cerebral palsy (CP) is the most common form of chronic motor disability in children. Other causes of disability in children include prolonged immobilization, nutritional factors, pubertal status and chronic use of anticonvulsants. All these factors may be related to impairment of normal bone development in these children.^(1)^ Low calcium intake is not uncommon in CP and may also contribute to poor mineralization of bone matrix.^(2)^ Consequently, children with severe CP are at risk for developing low bone mineral density (BMD) and low-impact fractures. However such a scenario could only be maintained after bone densitometry (dual-energy X-ray absorptiometry − DXA) became available,^(3)^ and the risk of fracture and low bone density in children is still not well defined when compared to adults.^(4)^ After its introduction, DXA remains the gold standard technique for the assessment of BMD in adults. This two-dimension measurement is also named as areal BMD (aBMD), and is influenced by bone size. This is an important problem in pediatric bone assessment because of the large differences in body size across different ages. The interpretation of bone mineral measurements is far more complex in children than in adults, since children are continuously growing. The relation between fracture risk and BMD measurements has not been well established in children. However, fractures and reduced bone mass secondary to disuse have been reported in several different diseases in children, and CP has been one of the most prevalent conditions seen in this group.^(5)^ Over the past 30 years, various studies have investigated BMD and fractures in children with CP and there is a considerable variation when assessed in the lumbar spine, distal femur or total body. According to International Society for Clinical Densitometry (ISCD) Pediatric Official Positions, children with chronic immobilization such as CP should have DXA scan to measure aBMD in spine and bone mineral content in total body.^(6-8)^

Pharmacological interventions have been used to ameliorate bone loss in children.^(9,10)^ Antiresorptives (bisphosphonates) associated to nutritional supplementation, vitamin D, calcium and weight-bearing physical activity program are known to be effective to improve BMD in these children. The recently developed antiresorptive RANKL antagonist, denosumab, decreases fracture incidence by increasing BMD and degree of mineralization in postmenopausal women. A faster and more profound inhibition of bone turnover by denosumab, when compared to oral bisphosphonates, has been observed as well as increasing in BMD at all sites. Denosumab inhibits osteclast-mediated bone resorption but works through a different pathway than bisphosphonates. This unique mechanism of action let us to therapeutically target RANKL in patients with CP and low bone mass,^(11,12)^ and to our knowledge this is the first report about children with CP treated with the RANKL antibody denosumab.

## OBJECTIVE

To assess the bone health status of children with cerebral palsy and the therapeutic effect of denosumab in a subgroup of children with cerebral palsy and decreased bone mass.

## METHODS

This study was carried at *Hospital Abreu Sodré*, São Paulo (SP), Brazil. This project was approved by the Ethics Committee in Research of the *Associação de Assistência à Criança Deficiente* under protocol number 120/2009. Our research group enrolled 183 children aged 5 to 14 years (mean age of 12 years). Children were classified according to the Gross Motor Functional Classification Scale (GMFCS). Severe CP was defined by a functional level of 4 or 5 on the GMFCS scale. Some children presenting moderate to severe CP, as per the GMFCS, were selected to receive denosumab. The initial evaluation included anthropometric measures and BMD assessment of the whole group. The BMD values of lumbar vertebrae (L1-L4) and total body, excluding the head, were determined by DXA (Lunar DPX - NT system, version 13.6, GE Healthcare). Since frequent motion artifact precluded reliable assessment of total body bone mass, we therefore relied on the Z-score of lumbar spine for evaluation. The children whose Z-score adjusted for age and sex was below -2.0 were classified as having low bone density for chronological age. The Z-score were provided by an appropriate software for pediatric patients.

C-terminal telopeptide of type 1 collagen (CTx) as a serum marker of bone resorption was measured, and the serum level of osteocalcin (OC) was determined to assess changes in bone formation. Both CTx and OC were measured by electrochemiluminescence (Roche Diagnostics). Parathyroid hormone (PTH), calcium, phosphorus, alkaline phosphatase and OH25 vitamin D were evaluated and, in all children, the values were within the normal range for age. The results were interpreted using reference data published by Orwoll et al. and Poomthavorn et al.^(12,13)^ Written informed consent from both parents of each patient was obtained. Ten parents randomly chosen agreed to bring the children for additional evaluation and were selected to receive denusomab 10mg subcutaneous. The statistical analyses were performed using the Wilcoxon paired *t*-test and Spearman correlation test.

## RESULTS

**Figure 1:**
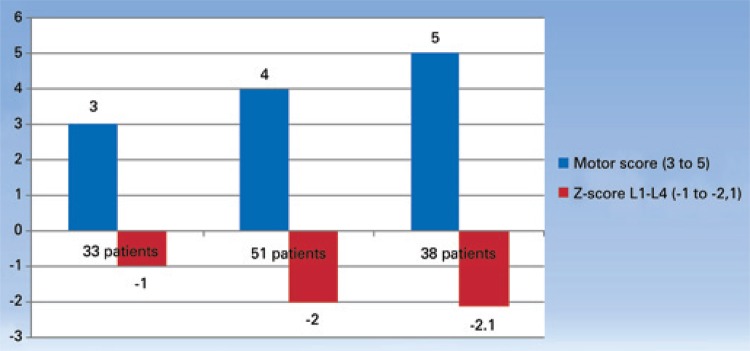
Correlation between motor score and Z-score at lumbar spine

**Figure 2:**
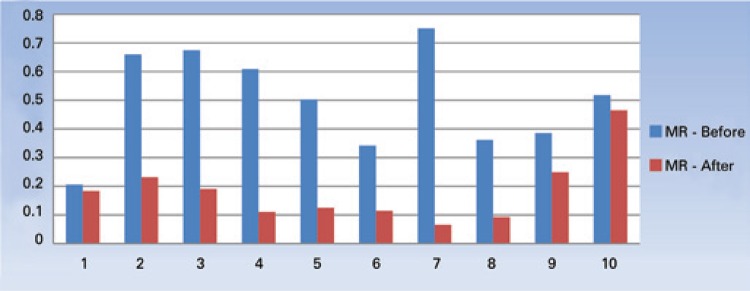
Bone turnover markers before and after denosumab injection: osteocalcin. Mean change from baseline in osteocalcin 3 months after denosumab injection. Bars show the 95% confidence interval p<0.05

A total of 161 children took part in the initial evaluation and performed DXA scan. From those, 39 were excluded because we did not obtain the GMFCS. Hence, 122 children (58 female and 64 male) aged 4 to 19 years were studied. Demographic, anthropometric, BMD and GMFCS data are displayed on tables 1 and 2. The correlation between motor score and bone mass is depicted on figure 1. A strong correlation was observed between score of motor deficiencies and decreased bone mass (p<0.01). Figure 2 shows the decreased bone markers after denusomab, evaluated three months after the single injection. A significant change was observed on both markers after denosumab administration (p<0.01). The denusomab injection was well tolerated by all ten patients with no adverse events.

**Table 1:** Descriptive statistics of anthropometrics characteristics, measures of central tendency and dispersion for bone mineral density and gross motor functional classification in palsy cerebral children (n=161)

Variable	Mean (SD)	Min	Max
Age, years	12.3 (0.7)	4	19
Weight, kg	28.4 (3.5)	10	70
Height, cm	129.1 (19.7)	179	80
BMI, kg/m^2^	17.7	12.5	25
Mineral density, z-score	-1.7 (1.0)	-5.2	1.1
GMFCS	3.9 (0)	0	5

SD: standard deviation; BMI: body mass index; GMFCS: Gross Motor Functional Classification Scale; Min: minimum; Max: maximum.

**Table 2:** Descriptive statistics of anthropometrics characteristics, measures of central tendency and dispersion for bone mineral density, Gross Motor Functional Classification Scale, bone turnover markers and vitamin D, in palsy cerebral children before receiving denusomab (n=10)

Child	Sex	Age (years)	Weight (kg)	Height (cm)	BMD (g/cm^**2**^)	z-score (SD)	GMFCS	OC	CTx	Vitamin D_(ng/mL)_
1	M	13	32	150	0.491	-3.1	5	18	0.206	12
2	M	12	27	129	0.364	-4.1	4	119.7	0.667	22.6
3	M	6	15	102	0.355	-3.7	5	40.6	0.674	11.2
4	F	10	10	90	0.351	-4.2	5	24.4	0.609	26.7
5	M	17	42	155	0.821	-3.0	4	31.2	0.503	12.4
6	M	5	10	92	0.332	-3.9	5	15.9	0.342	26.4
7	M	10	23	127	0.431	-3.1	4	19.7	0.751	21.2
8	M	7	16	110	0.417	-3.0	4	-	0.362	25.6
9	F	16	35	129	0.680	-4.1	4	33.9	0.386	-
10	M	13	35	150	0.606	-2.6	4	10.8	0.518	-

BMD: bone mineral density; SD: standard deviation; OC: osteocalcin; CTx: C-terminal telopeptide of type 1 collagen: M: male; F: female; GMFCS: Gross Motor Functional Classification Scale.

## DISCUSSION

Children with disabilities that limit mobility are at increased risk for osteoporosis. They often sustain fractures with minimal trauma that impair their function and quality of life. The growth in childhood and adolescence is critically important to produce healthy bone mass. Bone acquisition and remodeling are controlled by mechanical and metabolic factors. However, the acquisition of bone capital in patients with CP does not follow the normal pattern of the healthy population, because growth is slow in patients with CP and the BMD falls outside the normal range.

Medications approved for osteoporosis treatment include antiresorptive drugs, such as bisphosphonates, raloxifene, salmon calcitonin, RANKL antagonist, and one anabolic (bone-forming) drug, teriparatide − PTH 1-34.^(14)^

Despite the availability of many drugs and evidence demonstrating their efficacy to treat osteoporosis and reduce fracture risk in adults, only few drugs were used in children with very low bone density. Actually, there are very few studies in the literature evaluating pediatric osteoporosis treatment. Most studies available used bisphosphonates.

Our study is the first that evaluated the use of denosumab in children with CP. Our results showed a dramatic decrease (exception one case) in both CTx and OC serum concentration levels in the remainder children after three months of treatment with denosumab. Similar results were described by Semler et al., in a different pathological state.^(15)^ These authors reported the first use of denosumab in children with osteogenesis imperfecta I-VI. Their results showed that therapy was well tolerated, and the laboratory parameters showed that the denosumab treatment reversed the condition and reduced bone resorption. Interestingly, Boyce et al. described the use of denosumab in fibrous dysplasia, a rare skeletal disorder in which normal bone and bone marrow are replaced by fibro-osseous tissue, leading to fractures, functional impairment, deformity and pain.^(16)^

In this case, a young boy with a severe form of the disease was treated with denosumab due to an expanding femoral lesion with overexpression of RANKL. After seven months of treatment, a marked reduction in pain and bone turnover was observed, as well as reduced tumor growth rate. However, denosumab was associated with clinically significant disturbances of mineral metabolism, simultaneously during treatment and after discontinuation. After therapy withdrawal, there was rapid and dramatic rebound of bone turnover makers with CTx exceeding pre-treatment levels and accompanied by severe hypercalcemia.

Our research has some limitations. We still do not have bone turnover makers data six months after denosumab injection; therefore, we cannot predict how fast bone turnover makers levels will recover or not, which may indicate that the suppression of bone resorption by this drug may be reversible. Further, we did not evaluate other bone mineralization parameters. We cannot predict any disturbance of mineral metabolism, such as secondary hyperparathyroidism and hypophosphatemia, during denosumab treatment and severe hypercalcemia on discontinuation. These adverse events were described by Boyce et al. when treating the boy with fibrous dysplasia. Finally, it should be mentioned that CP has several factors that influence BMD, rather than osteoclastogenesis.^(16)^ Our results with denosumab should be confirmed in a larger series, taking into consideration the side effects described in adults, although in a minor scale, such as back and muscle pain.

## CONCLUSION

We found strong correlation between significant motor impairment and reduced bone mass in cerebral palsy patients. The unique mechanism of action of denosumab and its easy administration may play a role in improving low bone mineral density secondary to cerebral palsy.
