# Supplementary material for: Bone health in cerebral palsy and introduction of a novel therapy
Source: Einstein (Sao Paulo). 2015 Oct-Dec;13(4):555–9. doi: 10.1590/S1679-45082015AO3321 (PMC4878630; doi:10.1590/S1679-45082015AO3321)
Supplement: Translation

# Translation: A saúde do osso na paralisia cerebral e a introdução de uma nova terapia

**Authors:** Morton Aaron Scheinberg, Ricardo Prado Golmia, Adriana Maluf Elias Sallum, Maria Guadalupe Barbosa Pippa, Aline Pinheiros dos Santos Cortada, Telma Gomes da Silva

**Affiliations:** 1 Hospital Israelita Albert Einstein, São Paulo, SP, Brasil.; 2 Associação de Assistência à Criança Deficiente, São Paulo, SP, Brasil;; Lar Escola São Francisco, São Paulo, SP, Brasil.; 3 Associação de Assistência à Criança Deficiente, São Paulo, SP, Brasil.

**Keywords:** Paralisia cerebral/quimioterapia, Osteoporose/quimioterapia, Anticorpos monoclonais/uso terapêutico, Criança

## Abstract

**Objetivo:**

Avaliar o estado de saúde dos ossos em crianças com paralisia cerebral e o efeito terapêutico do denosumabe em um subgrupo de crianças com paralisia cerebral e redução da massa óssea.

**Métodos:**

Crianças com paralisia cerebral foram avaliadas de acordo com seu escore de incapacidade motora (sistema de classificação para funções motoras grossas, de III a V), e marcadores de *turnover* ósseo. Dual de absorção de energia de raios X foi utilizado para medir a coluna lombar e total do corpo menos cabeça. Posteriormente, um grupo de crianças com paralisia cerebral e osteoporose foi tratado com denosumabe, um anticorpo monoclonal totalmente humano. Marcadores de remodelação óssea foram medidos antes e três meses após o tratamento.

**Resultados:**

Houve uma redução da densidade óssea, particularmente em crianças com maior comprometimento do escore motor; os marcadores de remodelação óssea diminuíram em um grupo selecionado de crianças três meses depois de terem sido expostas ao denosumabe.

**Conclusão:**

A perda óssea esteve presente em crianças com importante comprometimento das funções motoras, além da redução nos níveis séricos de marcadores de reabsorção óssea com novos tratamentos.

## INTRODUÇÃO

A paralisia cerebral (PC) é a forma mais comum de incapacidade motora crônica em crianças. Outras causas de incapacidade em crianças incluem imobilização prolongada, fatores nutricionais, estado puberal e uso crônico de anticonvulsivantes. Todos esses fatores podem estar relacionados ao comprometimento do desenvolvimento ósseo normal nessas crianças.^(1)^ A baixa ingestão de cálcio não é incomum na PC e também pode contribuir para uma mineralização inadequada da matriz óssea.^(2)^ Consequentemente, as crianças com PC grave apresentam risco de ter baixa densidade mineral óssea (DMO) e fraturas por baixo impacto. No entanto, tal cenário só pôde ser traçado após o advento da densitometria óssea (densitometria por absorção de raios X de dupla energia − DEXA)^(3)^ e, mesmo assim, o risco de fratura e de baixa densidade óssea em crianças ainda não está bem definido, em comparação aos adultos.^(4)^ Desde sua introdução, a DEXA permanece a técnica padrão-ouro para avaliação da DMO em adultos. Essa medida bidimensional também é conhecida como DMO areal (DMOa) e é influenciada pelo tamanho do osso. Este é um problema importante na avaliação óssea pediátrica, devido às grandes diferenças do tamanho corporal em diferentes idades. A interpretação das medidas minerais ósseas é muito mais complexa em crianças do que em adultos, já que as crianças estão em constante crescimento. A relação entre o risco de fratura e as medidas de DMO ainda não foi bem estabelecida em crianças. No entanto, fraturas e redução da massa óssea secundária ao desuso têm sido relatadas em diversas doenças em crianças, e a PC é uma das doenças mais prevalentes nesse grupo.^(5)^ Nos últimos 30 anos, vários estudos investigaram DMO e fraturas em crianças com PC, e há uma variação considerável quando se avaliam a coluna lombar, o fêmur distal ou o corpo inteiro. De acordo com o Posicionamento Oficial Pediátrico da *International Society for Clinical Densitometry* (ISCD), as crianças com imobilização crônica como, por exemplo, PC, devem ser submetidas à DEXA para medir a DMOa na coluna, assim como o conteúdo mineral ósseo no corpo inteiro.^(6-8)^

Intervenções farmacológicas são utilizadas para melhorar a perda óssea em crianças.^(9,10)^ Drogas antirreabsortivas (bisfosfonatos) associadas a suplementos nutricionais, vitamina D, cálcio e programa de exercícios físicos com carga, são comprovadamente efetivos para melhorar a DMO nessas crianças. O denosumabe, um antireabsortivo antagonista do RANKL recentemente desenvolvido, diminui a incidência de fraturas, aumentando a DMO e o grau de mineralização em mulheres na pós-menopausa. O denosumabe comparado a bisfosfonatos orais, produz uma inibição mais rápida e profunda do *turnover* ósseo, além de um aumento da DMO em todos os sítios. O denosumabe inibe a reabsorção óssea mediada por osteoclastos, mas atua em uma via diferente da dos bisfosfonatos. Este mecanismo de ação única permite usar o RANKL como alvo terapêutico em pacientes com PC e baixa massa óssea^(11,12)^ e, até onde sabemos, este é o primeiro relato de crianças com PC tratadas com o anticorpo denosumabe contra RANKL.

## OBJETIVO

Avaliar o estado de saúde dos ossos em crianças com paralisia cerebral e o efeito terapêutico do denosumabe em um subgrupo de crianças com paralisia cerebral e redução da massa óssea.

## MÉTODOS

Este estudo foi realizado no Hospital Abreu Sodré, São Paulo (SP), Brasil. Este projeto foi aprovado pelo Comitê de Ética em Pesquisa da Associação de Assistência à Criança Deficiente, protocolo número 120/2009. Nosso grupo de pesquisa incluiu 183 crianças com idade entre 5 a 14 anos (média de 12 anos). As crianças foram classificadas de acordo com a escala *Gross Motor Functional Classification Scale *(GMFCS). PC grave foi definida como um nível funcional de 4 ou 5 na escala GMFCS. Algumas crianças com PC moderada a grave, conforme o GMFCS, foram selecionadas para receber denosumabe. A avaliação inicial incluiu medidas antropométricas e avaliação da DMO óssea de todo o grupo. Os valores de DMO das vértebras lombares (L1-L4) e do corpo inteiro, excluindo a cabeça, foram determinados por DEXA (sistema Lunar DPX - NT, versão 13.6, GE *Healthcare*). Como o artefato de movimento frequente impediu uma avaliação confiável da massa óssea corporal total, usamos o escore-Z da coluna lombar para fazer essa avaliação. As crianças cujo escore-Z ajustado para idade e sexo e ficou abaixo de -2,0 foram classificadas como tendo baixa densidade óssea para sua idade cronológica. Os escore-Z foram calculados por um *software *apropriado para pacientes pediátricos.

O telopeptídeo C-terminal do colágeno tipo 1 (CTx) foi medido como marcador sérico da reabsorção óssea, e o nível sérico de osteocalcina (OC) foi determinado para avaliar alterações na formação óssea. Tanto o CTx quanto a OC foram medidos por eletroquimioluminescência (*Roche Diagnostics*). Paratormônio (PTH), cálcio, fósforo, fosfatase alcalina, e 25-hidoxi vitamina D (25-OH vitamina D) foram testados e, em todas as crianças, estavam dentro da faixa de normalidade para a idade. Os resultados foram interpretados usando dados de referência publicados por Orwoll et al. e Poomthavorn et al.^(12,13)^ Foi obtido o consentimento informado por escrito de ambos os pais de cada paciente. Dez pais escolhidos aleatoriamente concordaram em trazer os filhos para uma avaliação adicional e foram selecionados para receber 10mg de denosumabe subcutâneo. Foram realizadas análises estatísticas com teste *t* pareado de Wilcoxon e teste de correlação de Spearman.

## RESULTADOS

O total de 161 crianças participaram da avaliação inicial e foram submetidas ao exame de DEXA. Dentre elas, 39 foram excluídas, pois não aplicamos o GMFCS. Assim, foram estudadas 122 crianças (58 meninas e 64 meninos) com idade entre 4 e 19 anos. Os dados demográficos, antropométricos, de DMO e GMFCS são apresentados nas tabelas 1 e 2. A correlação entre a escala motora e a massa óssea é ilustrada na figura 1. Observou-se uma forte correlação entre a escala de deficiência motora e diminuição da massa óssea (p<0,01). A figura 2 mostra uma redução nos marcadores ósseos após o uso de denosumabe, avaliada três meses após a injeção única. Houve uma alteração significativa em ambos os marcadores após a administração do denosumabe (p<0,01). A injeção de denosumabe foi bem tolerada por todos os dez pacientes sem nenhum evento adverso.

**Figura 1:** Correlação entre o escore motor e o escore-Z na coluna lombar

**Figura 2:** Marcadores de *turnover* ósseo antes e depois da injeção de denosumabe: osteocalcina. Mudança média nos níveis de osteocalcina, desde a medida inicial até 3 meses após a injeção de denosumabe. As colunas mostram o intervalo de confiança de 95% p<0,05

**Tabela 1:** Estatística descritiva das características antropométricas, medidas de tendência central e dispersão para densidade mineral óssea e classificação de funções motoras grossas para crianças com paralisia cerebral (n=161)

Variável	Média (DP)	Mín	Máx
Idade, anos	12,3 (0,7)	4	19
Peso, kg	28,4 (3,5)	10	70
Altura, cm	129,1 (19,7)	179	80
IMC, kg/m^2^	17,7	12,5	25
Densidade mineral óssea, escore-z	-1,7 (1,0)	-5,2	1,1
GMFCS	3,9 (0)	0	5

DP: desvio padrão; IMC: índice de massa corporal; GMFCS: *Gross Motor Functional Classification Scale*; Mín: mínimo; Máx: máximo.

**Tabela 2:** Estatística descritiva das características antropométricas, medidas de tendência central e dispersão para densidade mineral óssea e *Gross Motor Functional Classification Scale*, marcadores de *turnover* ósseo e vitamina D em crianças com paralisia cerebral antes de receber denosumabe (n=10)

Criança	Sexo	Idade (anos)	Peso (kg)	Altura (cm)	DMO (g/cm^2^)	Escore-Z (DP)	GMFCS	OC	CTx	Vitamina D_(ng/mL)_
1	M	13	32	150	0,491	-3,1	5	18	0,206	12
2	M	12	27	129	0,364	-4,1	4	119,7	0,667	22,6
3	M	6	15	102	0,355	-3,7	5	40,6	0,674	11,2
4	F	10	10	90	0,351	-4,2	5	24,4	0,609	26,7
5	M	17	42	155	0,821	-3,0	4	31,2	0,503	12,4
6	M	5	10	92	0,332	-3,9	5	15,9	0,342	26,4
7	M	10	23	127	0,431	-3,1	4	19,7	0,751	21,2
8	M	7	16	110	0,417	-3,0	4	-	0,362	25,6
9	F	16	35	129	0,680	-4,1	4	33,9	0,386	-
10	M	13	35	150	0,606	-2,6	4	10,8	0,518	-

DMO: densidade mineral óssea; DP: desvio padrão; OC: osteocalcina; CTx: telopeptídeo C-terminal do colágeno tipo 1; M: masculino; F: feminino; GMFCS:* Gross Motor Functional Classification Scale*.

## DISCUSSÃO

As crianças com deficiências que limitam a mobilidade apresentam um maior risco de osteoporose. Normalmente sofrem fraturas ao menor trauma, o que prejudica tanto a função quanto a qualidade de vida. As fases da infância e adolescência são extremamente importantes na produção de massa óssea saudável. A aquisição e a remodelação ósseas são controladas por fatores mecânicos e metabólicos. No entanto, a aquisição de capital ósseo em pacientes com PC não segue o padrão normal da população saudável, pois o crescimento em pacientes com PC é mais lento, e a DMO fica fora do intervalo normal.

Os medicamentos aprovados para tratamento da osteoporose incluem drogas antirreabsortivas, como bisfosfonatos, raloxifeno, calcitonina de salmão, antagonista de RANKL e um anabolizante (formador de osso), teriparatida − PTH 1-34.^(14)^

Apesar de existirem muitas drogas disponíveis com eficácia demonstrada no tratamento da osteoporose e redução do risco de fraturas em adultos, poucos medicamentos foram utilizados em crianças com densidade óssea muito baixa. Na verdade, há muito poucos estudos na literatura abordando o tratamento da osteoporose pediátrica. A maior parte dos estudos utilizou bifosfonatos.

Nosso estudo é o primeiro a avaliar o uso do denosumabe em crianças com PC. Os resultados mostraram uma diminuição drástica (exceto em um caso) nos níveis de concentração sérica de CTx e OC nas demais crianças após três meses de tratamento com denosumabe. Resultados semelhantes foram descritos por Semler et al., em um estado patológico diferente.^(15)^ Esses autores relataram o primeiro uso de denosumabe em crianças com osteogênese imperfecta I-VI. Os resultados mostraram que a terapia foi bem tolerada, e os parâmetros laboratoriais mostraram que o tratamento com denosumabe conseguiu reverter a situação e reduzir a reabsorção óssea. Curiosamente, Boyce et al., descreveram o uso do denosumabe na displasia fibrosa, uma doença esquelética rara em que o osso e a medula óssea normais são substituídos por tecido fibro-ósseo, levando ao aparecimento de fraturas, incapacidade funcional, deformidade e dor.^(16)^

Neste caso, um menino com uma forma grave da doença foi tratado com denosumabe para expansão da lesão femoral com superexpressão de RANKL. Depois de sete meses de tratamento, foi observada uma redução acentuada da dor e do *turnover* ósseo, bem como uma redução na taxa de crescimento do tumor. No entanto, o denosumabe foi associado a alterações clinicamente significativas do metabolismo mineral durante o tratamento e após a interrupção. Após a suspensão da terapia, houve recuperação rápida e drástica de marcadores de *turnover* ósseo com CTx, levando a valores superiores aos níveis pré-tratamento, acompanhada de hipercalcemia grave.

Nossa pesquisa teve algumas limitações. Ainda não temos os dados de marcadores de *turnover* ósseo de seis meses após a injeção de denosumabe e, por isso, não podemos prever com que velocidade os níveis de marcadores de *turnover* ósseo se recuperam ou não, o que indicaria que a supressão da reabsorção óssea promovida pelo medicamento pode ser reversível. Além disso, não avaliamos outros parâmetros de mineralização óssea. Não é possível prever transtornos do metabolismo mineral, como hiperparatireoidismo secundário e hipofosfatemia durante o tratamento com denosumabe, e hipercalcemia grave mediante a interrupção do medicamento. Esses efeitos adversos foram descritos por Boyce et al. quando trataram o menino com displasia fibrosa. Finalmente, é importante destacar que a PC envolve vários fatores que influenciam na DMO, além da osteoclastogênese.^(16)^Os resultados com denosumabe devem ser confirmados em uma casuística maior, considerando-se os efeitos colaterais descritos em adultos, embora em menor escala, como dor nas costas e dor muscular.

## CONCLUSÃO

Houve uma forte correlação entre comprometimento motor importante e redução da massa óssea em pacientes com paralisia cerebral. O mecanismo de ação único do denosumabe e sua fácil administração podem desempenhar um papel importante na melhora da baixa densidade mineral óssea secundária à paralisia cerebral.
